# Doxorubicin downregulates cell surface B7-H1 expression and upregulates its nuclear expression in breast cancer cells: role of B7-H1 as an anti-apoptotic molecule

**DOI:** 10.1186/bcr2605

**Published:** 2010-07-13

**Authors:** Hazem Ghebeh, Cynthia Lehe, Eman Barhoush, Khaldoon Al-Romaih, Asma Tulbah, Monther Al-Alwan, Siti-Faujiah Hendrayani, Pulicat Manogaran, Ayodele Alaiya, Taher Al-Tweigeri, Abdelilah Aboussekhra, Said Dermime

**Affiliations:** 1 Tumor Immunology Section, King Faisal Specialist Hospital and Research Center P.O. Box 3354, Riyadh 11211, Saudi Arabia; 2 Stem Cell Therapy Program, King Faisal Specialist Hospital and Research Center P.O. Box 3354, Riyadh 11211, Saudi Arabia; 3 Department of Pathology, King Faisal Specialist Hospital and Research Center P.O. Box 3354, Riyadh 11211, Saudi Arabia; 4 Department of Biological and Medical Research, King Faisal Specialist Hospital and Research Center P.O. Box 3354, Riyadh 11211, Saudi Arabia; 5 Oncology Center, King Faisal Specialist Hospital and Research Center P.O. Box 3354, Riyadh 11211, Saudi Arabia; 6 Senior Scientist, Chairman of Department of Biomedical Research, Head of Immunology and Innovative Cell Therapy Unit, Dasman Diabetes Institute, PO Box 1180, Dasman 15462, Kuwait

## Abstract

**Introduction:**

B7-H1 (PD-L1, CD274) is a T cell inhibitory molecule expressed in many types of cancer, leading to immune escape of tumor cells. Indeed, in previous reports we have shown an association of B7-H1 expression with high-risk breast cancer patients.

**Methods:**

In the current study, we used immunohistochemistry, immunofluorescence and Western blot techniques to investigate the effect of neoadjuvant chemotherapy on the expression of B7-H1 in breast cancer cells.

**Results:**

Among tested chemotherapeutic agents, doxorubicin was the most effective in downregulating cell surface expression of B7-H1 *in vitro*. These results were validated *in vivo *in a xenograft mouse model, as well as in murine heart tissue known to constitutively express B7-H1. The doxorubicin-dependent cell surface downregulation of B7-H1 was accompanied by an upregulation of B7-H1 in the nucleus. This re-distribution of B7-H1 was concurrent with a similar translocation of phosphorylated AKT to the nucleus. Inhibition of the PI3K/AKT pathway abrogated the doxorubicin-mediated nuclear up-regulation of B7-H1, suggesting an involvement of PI3K/AKT pathway in the nuclear up-regulation of B7-H1. Interestingly, siRNA knock down of B7-H1 lead to an increase in spontaneous apoptosis, as well as doxorubicin-induced apoptosis, which indicates an anti-apoptotic role for B7-H1 in breast cancer cells. The novel discovery of B7-H1 expression in the nuclei of breast cancer cells suggests that B7-H1 has functions other than inhibition of T cells.

**Conclusions:**

Our findings explain the previously reported immunomodulatory effect of anthracyclines on cancer cells, and provide a link between immunoresistance and chemoresistance. Finally these results suggest the use of dual combinatorial agents to inhibit B7-H1 beside chemotherapy, in breast cancer patients.

## Introduction

Anthracyclines rank amongst the most effective anti cancer drugs ever developed [1]. Whereas doxorubicin is an essential component of treatment for breast cancer [2], childhood solid tumors, soft tissue sarcomas and aggressive lymphomas [3,4], daunorubicin shows activity in acute lymphoblastic or myeloblastic leukemias [5]. Like many chemotherapeutic drugs, anthracyclines kill cancer cells by direct cytotoxicity. Nevertheless, there is accumulating evidence that these agents also have immuno-augmenting effects, through both the innate as well as the adaptive immune system, that might help in the therapy of cancer [6]. Doxorubicin stimulates cytokines production, augments natural killer (NK) cells activity [7], stimulates cytotoxic T-lymphocyte (CTL) responses [8] and augments differentiation of macrophages [9], all of which are important components of an effective immune response. Recently, the unique ability of doxorubicin, daunorubicin and mitoxantrone to make cancer cells immunogenic was shown to be through calreticulin re-localization to the cell surface [10] and the selective induction and release of High-mobility group box 1 (HMGB1) protein from dying cancer cells [11].

The mainstay of the adaptive immune system is the antigen presentation of processed peptides by antigen presenting cells (APC) [12,13]. Recognition of a T cell receptor of a peptide presented on MHC molecules of an APC provides the first signal. However, the optimal activation of a T lymphocyte requires a second signal provided by co-stimulatory molecules, which are normally balanced with inhibitory molecules [14]. The balance of positive and negative signals is of central importance in maximizing the ability of the adaptive immune response to defend the host, while maintaining tolerance and preventing autoimmunity [15]. One of the recently identified novel T lymphocyte inhibitory molecules is the cell surface glycoprotein called B7-H1 (also called PD-L1 and CD274). B7-H1 is expressed on APCs and binds to its ligand on T lymphocytes leading to both inhibition and induction of apoptosis in effector T lymphocytes [15], or induction of anergy in naïve T lymphocytes [16-18].

The aberrant expression of B7-H1 in tumor tissues has been reported in various cancers [19]. Our group has recently reported on the aberrant B7-H1 expression in breast cancer tissues and its association with high-risk prognostic factors [20]. In the current study we examine the effect of chemotherapeutic agents, commonly used for treatment of breast cancer, on the expression level of B7-H1 in breast cancer cells. We have shown doxorubicin-dependent downregulation of cell surface B7-H1 and its translocation to the nucleus concomitant with the translocation of the phospho-AKT. Finally, we provide evidence that B7-H1 has an anti-apoptotic role in doxorubicin-treated breast cancer cells.

## Materials and methods

### Drug treatment of cultured cell lines and AKT phosphorylation inhibition

MDA-MB-231, SKBR-3 and T47 D cells (ATCC) were cultured in Dulbecco's Modified Eagle's Medium (DMEM) with 10% FCS. Cells were seeded at 2 × 10^4 ^cells/cm^2^. Doxorubicin, Daunorubicin, Mitoxantrone, Cisplatin (Sigma, St. Louis, MO, USA) and Docetaxel (Aventis Pharma, Bridgewater, NJ, USA) were added to the cells at 60 to 80% confluence. Cells were incubated with the drug for 24 to 72 hours. AKT phosphorylation was inhibited using LY294002 (calbiochem) at 20 μM.

### Measurement of B7-H1 expression and apoptosis

B7-H1 expression was assessed by FACS analysis of allophycocyanin-labeled anti-B7-H1 (eBioscience, San Diego, CA, USA) or Phycoerythrin -labeled B7-H1 (eBioscience) to eliminate the interference of the natural fluorescence of the drug [21,22]. Cells were incubated with anti-B7-H1 for 45 minutes before FACS analysis. Cell-viability was measured using 1.5 μg/mL propidium iodide. For the apoptosis assay, cells were doublestained with anti-B7-H1 antibody and Annexin V (Apoptosis Assay Kit system, Molecular Probe, Eugene, OR, USA). luorescence was measured using LSR I FACS system (BD Biosciences, San Jose, CA, USA) and analyzed using Cell Quest Pro analysis software (BD Biosciences).

### Measurement of cell proliferation

MDA-MB-231 cells were cultured in a 96-well plate (10,000 cells/well) for 24 hours followed by the addition of ^3^H-thymidine (Amersham, Chiltern Hills, London, UK) at 1 μCi/well for 18 hours before harvesting. ^3^H-thymidine uptake was measured using a 1450 Micro Beta PLUS liquid scintillation counter (Wallac, Waltham, Massachusetts, USA).

### Western blot analysis

Cells were lysed and cell membrane proteins were separated using a membrane protein extraction kit (Biovision, Mountain View, California, USA). Cytoplasmic and nuclear protein extracts were prepared as previously published [23]. Immunoblotting was performed as described previously [24]. Anti-B7-H1 (MIH1 clone, eBioscience) was used at 1:500 dilutions and Anti-GAPDH (FL-335 clone, Santa Cruz, Santa Cruz, CA, USA) was used at 1:4000 dilution.

### Immunofluorescence

MDA-MB-231 cells cultured on collagen type I (BD Biosciences) coated slides were treated with doxorubicin for 72 hours. The slides were washed, dried and fixed in acetone. Cells were stained overnight with anti-B7-H1 at 1:50. Cells were washed and stained with biotinylated polyclonal antibody (Ultratech, San Jose, CA, USA) for 30 minutes. After washing, streptavidin- fluorescein isothiocyanate (FITC) (BD Biosciences) was added at 1:100 for 30 minutes. Phosphorylated AKT (phospho-AKT) staining was carried out by fixing cells in 1% paraformaldehyde, washing in permeabilization buffer (eBioscience) and one-hour incubation with rabbit anti-phospho-AKT (Ser473 site, Cell Signaling, Danvers, MA, USA). After washing, cells were incubated with FITC-goat-anti rabbit-antibody (Serotec, Raleigh, NC, USA) at 1:10 for 30 minutes. 300 nM DAPI (Invitrogen, Carlsbad, CA, USA) was added for 10 minutes to counterstain the nuclei. Slides were mounted and immunofluorescence was visualized using confocal microscope (Perkin Elmer Ultraview, Covina, CA, USA). Cytoplasmic and nuclear AKT were quantified with manual counting from printed images.

### Mice xenotransplantation and drug treatment

Nude mice (Jackson Laboratories, Bar Harbor, Maine, USA) were injected with 10^6 ^cells of MDA-MB-231 in the mammary pad and tumor size was measured weekly using a caliper. When the tumor reached approximately 7 mm diameter (four to five weeks), mice were treated with doxorubicin (10 mg/Kg) via tail vein injection. After three or five days, the mice were sacrificed and tumor and heart tissues were excised, embedded in OCT, snap frozen in liquid nitrogen and stored at -80°C before use. The breeding, care and sacrifice of the animals were in accordance with the protocols approved by the Animal Care and Use Committee of the King Faisal Specialist Hospital and Research Centre.

### Immunohistochemistry

Mouse tumor and heart tissues were stained using rabbit anti-B7-H1 (LifeSpan BioSciences, Seattle, WA, USA) at 1:2000 followed by HRP-Envision anti-rabbit-polymer (Dako, Carpinteria, CA, USA) as previously described [25]. Color was visualized with DAB (Ultratech) and nuclei were counterstained with instant hematoxylin (Shandon, Bohemia, NY, USA).

### B7-H1 siRNA treatment of cells

B7-H1 expression was inhibited in MDA-MB-231 cells using a specific siRNA (CD274 siRNA ID = s26547 and siRNA ID = s26548, Ambion, Austin, TX, USA). During siRNA transfection, cells were inoculated at a density of 20 × 10^3 ^cells/cm^2 ^in OPTI-MEM medium (GIBCO, Grand Island, NY, USA). Cells were allowed to grow for two days before drug treatments.

### Statistical analysis

Significance for B7-H1 expression in Neoadjuvant treated and non-treated patients were calculated using unpaired T-test using GraphPadPrism4 software, GraphPad Software, Inc., La Jolla, CA, USA). *P *< 0.05 cut-off was used to indicate significance.

## Results

### Doxorubicin down-regulates cell surface expression of B7-H1 *in vitro*

**Figure 1:**
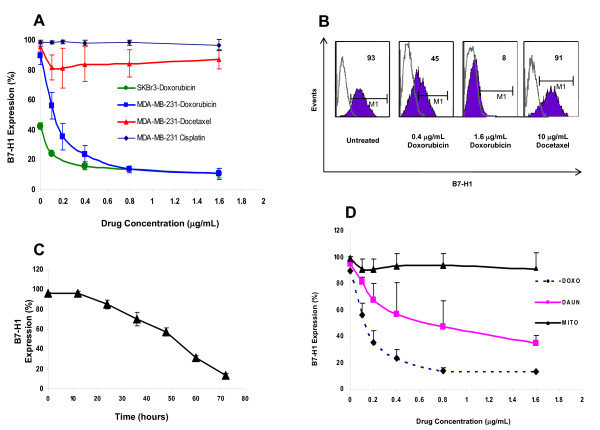
**The effect of chemotherapy on B7-H1 expression in breast cancer cell lines**. **A) **Dose dependent effect of chemotherapy in MDA-MB-231 and SKBr3 cells treated for 72 hours (n = 3). **B) **Representative FACS histograms for the effect of doxorubicin on MDA-MB-231 cells. **C) **Time dependent effect of doxorubicin (0.8 μg/mL) on MDA-MB-231 cells (n = 3). **D) **The Effect of different drugs on B7-H1 expression in MDA-MB-231 cells treated for 72 hours (n = 2). Doxo, doxorubicin; DAUN, daunorubicin; and MITO, mitoxantrone.

Currently, our standard neoadjuvant chemotherapy for locally advanced breast cancer patients involves doxorubicin, docetaxel and cyclophosphamide with some of the clinical trial enrolled patients receiving a combination of doxorubicin and cisplatin [26]. Cyclophosphamide being a prodrug, that is, an inactive compound that becomes activated only *in vivo*, we only tested the other three drugs on the expression of B7-H1. We used the two breast cancer cell lines (MDA-MB-231 and SKBr3) known to constitutively express B7-H1. Figure 1A, B shows a dose-dependent effect of these drugs on the cell surface expression of B7-H1. Doxorubicin treatment significantly down regulates B7-H1 expression, by approximately 80% at 0.4 ug/ml (Figure 1A). On the other hand docetaxel and cisplatin had no effect on the B7-H1 cell surface expression (see Figure 1A). The effect of doxorubicin on B7-H1 downregulation was time dependent reaching a 90% inhibition after 72 hours incubation (Figure 1C). In order to determine if the effect of Doxorubicin is shared by other members of the anthracycline family we tested the effect of daunorubicin, another member of the anthracyclines family which was found to have a strong downregulatory effect on the cell surface expression of B7-H1. Mitoxantrone, an immunomodulatory drug, had no significant effect on B7-H1 expression (Figure 1D).

### Doxorubicin-dependent cell surface downregulation of B7-H1 expression is not due to apoptosis or cell proliferation

**Figure 2:**
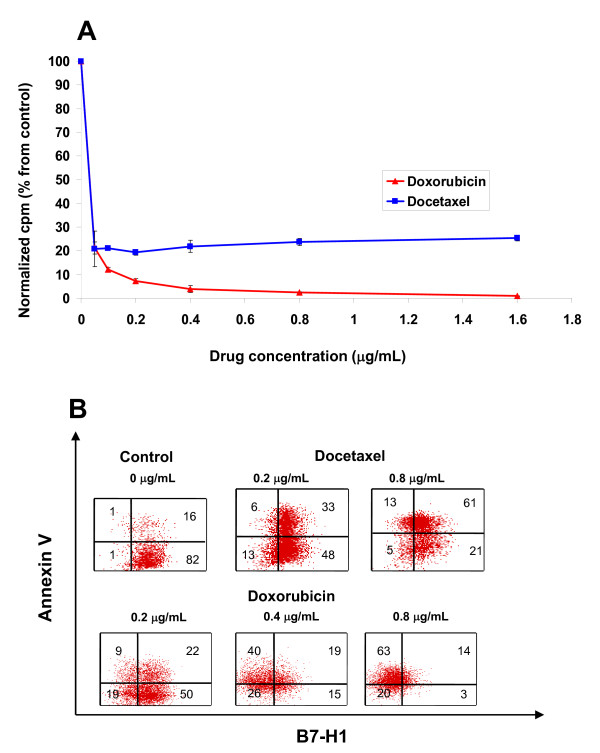
**The effect of chemotherapy on the survival and proliferation of cells**. **A) **The Effect of doxorubicin and docetaxel on the proliferation of MDA-MB-231 after 72 hours culture using ^3^H-thymidine uptakes. Counts (cpm) were normalized to untreated control and expressed as percentage of control. **B) **B7-H1 expression after doxorubicin treatment in apoptotic and non-apoptotic cells. Cells were treated with doxorubicin or docetaxel for 72 hours, doublestained with annexin V and B7-H1 and analyzed by FACs.

One of the known mechanisms of chemotherapeutic agents is to arrest the cell cycle thus stopping the proliferation of cells. Previously, we showed an association between B7-H1 expression and cell proliferation [27]. Therefore, we investigated whether the effect of doxorubicin on decreasing the B7-H1 was due to its effect on the proliferation by comparing the effect of docetaxel and doxorubicin on cell proliferation. Figure 2A shows that both drugs stopped MDA-MB-231 cell proliferation at the lowest tested concentration (0.05 ug/mL). However, B7-H1 expression is hardly effected at this concentration as shown in Figure 1A.

It is commonly known that apoptosis induces several changes in the cell membrane leading to redistribution of phosphatidylserine to the extra-cellular surface [28] and probably also other surface molecules. To test whether such a decrease in the cell surface expression of B7-H1 is due to apoptotic cell membrane redistribution of membrane molecules, we double-stained for B7-H1 and annexin V, a molecule that binds to negatively charged phosphatidylserine and thus labels apoptotic cells. Figure 2B shows MDA-MB-231 cells treated with doxorubicin or docetaxel for 72 hours and double-labeled with B7-H1 and Annexin V. Doxorubicin downregulated B7-H1 in both apoptotic and non-apoptotic cells. Interestingly, doxorubicin treatment resulted in a significant increase in apoptosis, mainly in the B7-H1 negative population. Furthermore, while both drugs induce apoptosis in a dose dependent manner, only doxorubicin downregulated B7-H1 expression, indicating that B7-H1 cell surface downregulation is independent of membrane redistribution of phospholipids. Altogether these results suggest that the downregulatory effect of doxorubicin on the surface expression of B7-H1 is specific and not due to apoptosis mediated membrane re-distribution of phospholipids or blockage of cell proliferation.

### Downregulation of cell surface expression of B7-H1 after doxorubicin treatment is due to cellular redistribution

**Figure 3:**
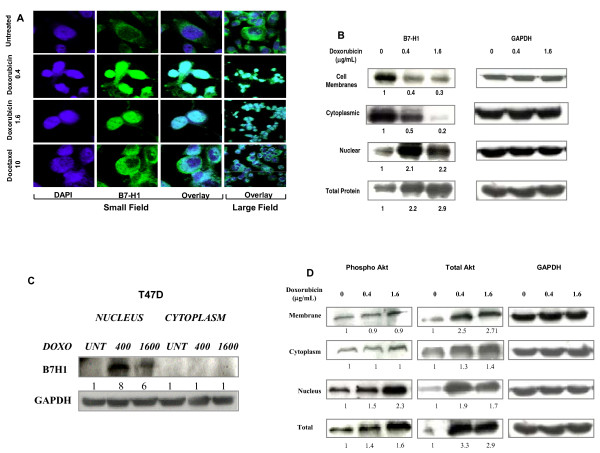
**The effect of doxorubicin on B7-H1 cellular distribution**. **A) **Representative confocal immunofluorescence image of MDA-MB-231 cells monolayer after drug treatments. The overlay of B7-H1 (green) DAPI (nucleus blue) is shown as bluish white color. Numbers beside drugs indicates the concentration in μg/mL. **B **and **D) **Western blot showing B7-H1 and phospho-AKT expression respectively in different protein fractions of MDA-MB-231 cells treated with doxorubicin for 72 hours. **C) **Western blot showing B7-H1 expression in different protein fractions of T47 D cells treated with doxorubicin for 72 hours. GAPDH was used as control as well as for quantification of the B7-H1 expression.

The above data demonstrated a downregulation of cell surface expression of B7-H1 in breast cancer cells after treatment with doxorubicin as assessed by flow cytometry. To investigate the mechanism of this downregulation, we measured the B7-H1 mRNA using RT-PCR of the total RNA collected from cells treated with two doses of doxorubicin (0.4 and 0.8 μg/mL). There was no significant change in the level of the B7-H1 mRNA (data not shown). We further studied the redistribution of the B7-H1 protein into the cells by staining a monolayer of MDA-MB-231 cells with immunofluorescence labeled B7-H1 and examining the cells under confocal microscope. Untreated cells had membranous/cytoplasmic expression of B7-H1 with predominant staining in the cytoplasm close to the nuclear membrane, possibly the endoplasmic reticulum. In the nucleus, a very low expression of B7-H1 could be seen in the small field image (Figure 3A). However, after treatment with 0.4 μg/mL doxorubicin, the expression of B7-H1 was more localized to the nuclei (in more > 95% of the cells) with some expression in the cytoplasm. At 1.6 μg/mL of doxorubicin there was a predominant expression of B7-H1 in the cell nuclei. Consistent with the flow cytometry data, docetaxel did not decrease the cell surface expression of B7-H1 (Figure 3A). To further confirm B7-H1 redistribution after treatment we extracted proteins from the membrane, cytoplasm and the nucleus of the cells and measured B7-H1 expression in these fractions using western blot before and after treatment. Figure 3B shows a three-fold decrease in the level of B7-H1 in the membrane and a five-fold decrease in the level of B7-H1 in the cytoplasmic fractions However there was a two-fold increase in the level of B7-H1 in the nuclear fraction after doxorubicin treatment. These results were also seen with other cell lines that express very low levels of B7-H1 on the cell surface like T47 D (Figure 3C). Altogether the confocal microscopy and the Western blot data clearly demonstrated a doxorubicin-dependent re-distribution of B7-H1 from the membrane to nucleus.

### Re-distribution of B7-H1 from the membrane to the nucleus is associated with translocation of p-AKT to the nucleus

Several reports have previously shown a direct relationship between the AKT activation pathway and B7-H1 expression, and these studies demonstrated that B7-H1 is a downstream target of AKT in glioma and breast cancer cells [29,30]. In the present study, we measured phospho-AKT and total AKT after doxorubicin treatment of MDA-MB-231 cells using Western blot analysis. There was no change in phospho-AKT levels in the membrane and cytoplasm fractions; however, there was an increase in total AKT in these fractions resulting in a decrease of phospho-AKT over total AKT (Figure 3D). In contrast, there was an increase in both nuclear phospho-AKT and total AKT. Total cell proteins show a net increase in both phospho-AKT and total AKT (Figure 3D). The decrease of phospho-AKT in the membrane and cytoplasm and its upregulation in the nucleus is associated with B7-H1 re-distribution after doxorubicin treatment.

### B7-H1 inhibition increases the apoptotic effect of doxorubicin in breast cancer cells

**Figure 4:**
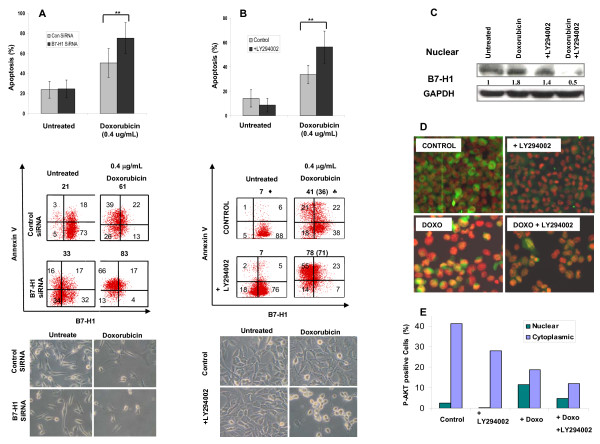
**The effect of doxorubicin on B7-H1 and AKT expression and induction of apoptosis**. **A) **Column chart of the FACS data showing the effect of siRNA-B7-H1 inhibition on the percentage of apoptosis induced after 48-hours treatment of MDA-MB-231 cells with doxorubicin (n = 6, **indicated *P *< 0.001) (Top panel), representative FACS data of one of the experiments (middle panel) and image of the cells right before harvesting (bottom panel). **B) **Column chart of the FACS data showing the effect of specific AKT-inhibitor (LY294002) on B7-H1 expression and induction of apoptosis in MDA-MB-231 cells after 48 hours doxorubicin treatment (n = 5, **indicates *P *= 0.016) (Top panel), representative FACS data of one of the experiments (middle panel) Diamond, total percentage of apoptotic cells, Spade, numbers in brackets are specific doxorubicin induced apoptosis (SDA) calculated by subtracting the percent of apoptotic cells in non-treated control. Image of the cells right before harvesting is indicated in the bottom panel. (The columns are means and error bars are SEMs in the top panel of A and B). **C) **Western blot for nuclear proteins from the same cells as in B stained with B7-H1 or GAPDH. **D) **Representative immunofluorescence images of the same cells as in B stained with a specific antibody for phospho-AKT (green). **E) **Cytoplasmic and nucleus AKT were quantified manually from the presented image shown in D. Nuclei were counterstained with DAPI (Red).

Recently, cell surface B7-H1 has been demonstrated to play an anti-apoptotic role in a mouse cancer model [31]. Other studies showed that phospho-AKT, as an anti apoptotic molecule, is upstream of B7-H1 [29,30]. Therefore, we investigated whether the presence of B7-H1 plays a role in apoptosis. In the present study, we investigated its possible anti-apoptotic role in human breast cancer cells. We inhibited B7-H1 using a specific siRNA before treating MDA-MB-231 cells with doxorubicin. B7-H1-siRNA treatment resulted in downregulation of B7-H1 cell surface expression from 91% to 49% (Figure 4A middle). Interestingly, there was an increase in apoptosis from 50 ± 14% in control-siRNA treated to 75 ± 15% in B7-H1-siRNA transfected cells after doxorubicin treatment (*P *< 0.001). This translates into a 1.5-fold increase in the specific doxorubicin induced apoptosis (SDA) in the B7-H1 negative cells treated with B7-H1-siRNA inhibitor (Figure 4A top) indicating an anti-apoptotic role of B7-H1 in breast cancer cells (the columns are means and error bars are SEMs). Similar results were obtained using a different B7-H1-siRNA (CD274: siRNA ID = s26548) inhibitor (Additional file 1) confirming the specific inhibition effect of B7-H1. Cell shrinkage, a typical feature of apoptosis is shown at the bottom of Figure 4A.

### The anti-apoptotic role of B7-H1 is PI3K/AKT pathway dependent

Because B7-H1 is a downstream target of AKT, we investigated whether the anti-apoptotic effect of B7-H1 is associated with the AKT pathway. We tested the effect of inhibiting AKT phosphorylation using PI3K/AKT inhibitor (LY294002) on B7-H1 expression and function. Interestingly, the inhibition of the AKT pathway resulted in a two-fold increase in the specific doxorubicin induced apoptosis (*P *= 0.016) (Figure 4B top). The columns are means and error bars are SEMs. The effect of inhibiting PI3K/AKT pathway on B7-H1 expression was also investigated. While AKT Inhibition partially decreased cell surface B7-H1 expression, doxorubicin treatment of the inhibited cells synergistically decreased cell surface B7-H1 expression (from 60 to 30%) (Figure 4B middle). Cell shrinkage, a typical feature of apoptosis is shown at the bottom of Figure 4B. As shown in Figure 3B there was an upregulation of B7-H1 expression in the nucleus of doxorubicin treated cells. However, doxorubicin treatment of PI3K-inhibited cells had a significant low expression of the nuclear B7-H1 (Figure 4C). The increase of the nuclear B7-H1 in PI3K-inhibited cells, in the absence of doxorubicin treatment, may be due to a reverse signal of B7-H1 as it has been suggested recently [32]. Our data suggest that doxorubicin-dependent upregulation of B7-H1 in the nucleus is in part dependent on the AKT pathway. To confirm our finding for the translocation of phospho-AKT to the nucleus, we stained the cells with an antibody-specific for phospho-AKT. Doxorubicin redistributed phospho-AKT from the membrane and cytoplasm to the nucleus consistent with the B7-H1 nuclear translocation (Figure 4D, E). Overall these results show that doxorubicin upregulates B7-H1 in the nucleus via an AKT-dependent mechanism to oppose the apoptosis of the cells. On the other hand, doxorubicin downregulates B7-H1 from the cell membranes through an unknown, AKT-independent pathway.

### Doxorubicin decrease cell surface expression of B7-H1 *in vivo*

**Figure 5:**
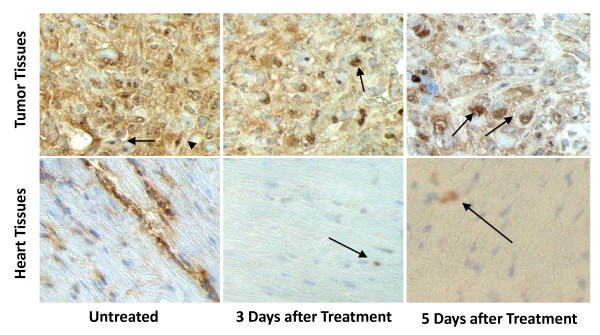
**Effect of doxorubicin on the *in vivo *expression of B7-H1**. Representative Immunohistochemical images (× 540) for B7-H1 (brown) expression in doxorubicin-treated and untreated mice. Shown are sections for tumors formed from xenotransplanted MDA-MB-231 cells in nude mice as well as heart tissues of the nude mice. Nuclei are counterstained with a light hematoxylin to show the nuclear B7-H1 expression. Arrows indicates the nuclear staining of B7-H1.

To confirm our *in vitro *observation of doxorubicin downregulation of B7-H1 in an *in vivo *system, we used a mouse model where we xenotransplanted MDA-MB-231 in nude mice in the mammary fat pad to closely mimic human breast cancer. When tumors reached 7 mm some of the mice were not treated (n = 2), while others (n = 2) were treated with doxorubicin at 10 mg/Kg. Tumor tissues from treated and untreated mice were surgically removed. In addition, we removed the heart as heart cells are known to constitutively express B7-H1 [15]. The tumor tissues had lower expression of B7-H1 after doxorubicin treatment as shown in Figure 6. Interestingly, the nuclear expression of B7-H1 was clear after doxorubicin treatment. Remarkably, there was a significant decrease in B7-H1 expression in the heart tissues of the treated mice where most heart myocytes were negative for B7-H1 staining and very few nuclear B7-H1 expressions could be seen (Figure 5). In conclusion, doxorubicin downregulates surface expression of B7-H1 in tumor cells as well as normal heart tissue *in vivo*, and triggers its re-distribution into nuclei confirming the physiological relevance of our *in vitro *findings.

## Discussion

The unique ability of anthracyclines to induce immunogenic tumor cell death has triggered many studies in order to understand the immunomodulatory effect of this family of chemotherapeutic agents. However, to our knowledge, the effect of anthracyclines on co-stimulation has never been investigated, despite its central role in the adaptive immune system. B7-H1 is a negative co-stimulatory molecule that is expressed in many cancers, where it is believed to contribute to the escape of tumors from immune recognition [33]. In this study, we demonstrated doxorubicin-mediated downregulation of B7-H1 surface expression and its localization to the nucleus.

We have previously reported the aberrant expression of B7-H1 in breast cancer tissues from 69 patients, some of which received chemotherapy while others did not [20,25,27]. However, we were not able to draw a conclusion on the effect of chemotherapy on the expression of B7-H1 in the corresponding tissues due to several factors. First, the tissue samples were taken after surgery, which is typically scheduled weeks after chemotherapy treatment, giving time for patient's blood counts to normalize. Additionally, the patients received a cocktail of chemotherapeutic agents which could have different effects on B7-H1 expression. Therefore, in this current study we took an *in vitro *approach to further test the specific effect of different chemotherapeutic agents on B7-H1 expression. We then confirmed our results *in vivo *mouse models where samples can be taken as soon as three to five days after treatment.

Doxorubicin, as one of the most effective anthracyclines drugs, specifically decreased B7-H1 expression in two cell lines known to constitutively express B7-H1 (MDA-MB-231 and SKBr3), while the other chemotherapeutic agents (cisplatin and docetaxel) did not have any significant effect on B7-H1 expression. It is important to note that the modulation of B7-H1 was observed at clinically relevant concentrations [34]. Interestingly, the effect of doxorubicin on B7-H1 expression was more prominent than daunorubicin. This is consistent with previous reports which indicate that doxorubicin has more immunogenic effect on cancer cells than daunorubicin [35]. On the other hand, mitoxantrone, which is remotely related to other anthracyclines, had no significant effect on B7-H1 cell surface expression. This may be due to the lack of the daunosamine sugar moiety and a substituted aglyconic anthraquinone [36]. Many investigators reported an effect of chemotherapeutic agents on co-stimulatory molecules such as B7.1. For example, Vereecque *et al *[37] reported an increase in B7.1 expression in Da1-3b leukemic cells following treatment with Ara-C and a slight decrease in B7-H1 expression. Similarly, the Mokyr group [38] reported upregulation of B7.1 and B7.2 molecules after treatment with L-PAM in MOPC-315 tumor bearing mice. However, Zhang *et al *[39] reported a negative co-stimulatory effect of different groups of chemotherapeutic agents, namely paclitaxel and etoposide, in breast cancer cells due to the upregulation of B7-H1. This suggests that different chemotherapeutic agents may have different effects on co-stimulatory molecules. In this regard, it is also important to note that none of the previous reports studied anthracyclines' effects on co-stimulation. Anthracyclines are unique among many chemotherapeutic agents in their ability to induce immunogenic apoptotic death in cancer cells. This has clearly been established by others who demonstrated that among various chemotherapeutic agents tested, anthracyclines were the only effective agents that provided mice with enhanced immunity when further challenged with tumor cells [10,40]. The effect of doxorubicin on B7-H1 reported in this study might, at least partially, explain doxorubicin's ability to make tumor cells immunogenic beside the other above discussed mechanisms.

Many chemotherapeutic agents, including doxorubicin and docetaxel, kill cancer cells through apoptosis, a process of cell death that is accompanied by cell membrane flip-flop leading to phosphatidylserine exposure, cell shrinkage and bleb formation; yet the cell membrane remains intact. The decrease of B7-H1 expression observed in the present study was not due to apoptotic membrane flipping. Moreover, docetaxel, which induced apoptosis in large numbers of cells, had no significant effect on B7-H1 expression demonstrating the specific effect of doxorubicin on B7-H1 surface downregulation.

In a mouse model, it has been shown that B7-H1 cell surface expression has an anti-apoptotic effect, where it is described as a *molecular shield *to protect cells from apoptosis [31]. Using specific B7-H1-siRNA, we have knocked down B7-H1 and have shown that doxorubicin induced more significant apoptosis, indicating a possible anti-apoptotic role for B7-H1 in breast cancer cells. However, the precise mechanism by which B7-H1 protects cells from apoptosis is unknown.

This is the first report to demonstrate the presence of nuclear expression of B7-H1 in breast cancer cells and its upregulation after drug treatment. The nuclear localization of B7-H1 suggests a function that extends beyond its role in inhibiting T lymphocytes. Our observation, that doxorubicin upregulates B7-H1 specifically in the nucleus and the significantly enhanced apoptosis after following a combination of B7-H1 knockdown and doxorubicin treatment, led us to speculate that the anti-apoptotic function of B7-H1 is due to its nuclear localization. The nuclear translocation of B7-H1 might allow its interaction with the apoptotic machinery of cells to regulate apoptosis. It has been reported that the nucleolus contains many anti-apoptotic molecules that promote cell survival after exposure to stress (Reviewed in [41]). At the clinical level, these findings might encourage targeting B7-H1 expression in conjunction with doxorubicin treatment.

The upregulation of phospho-AKT in the nucleus following doxorubicin treatment has been reported previously [42,43]. A subsequent study revealed an enhancement of apoptosis after PI3K/AKT pathway inhibition [44]. Using Western blot and immunofluorescence assays, we showed concurrent B7-H1 and phospho-AKT translocation to the nucleus. Furthermore, we demonstrated a similar effect of AKT and B7-H1 as anti-apoptotic molecules. Most importantly, B7-H1 up-regulation was inhibited using the PI3K/AKT inhibitor LY294002. This suggests that B7-H1 is upregulated in the nucleus via an AKT-dependent pathway. This is supported by previous studies demonstrating that B7-H1 is downstream of the PI3K/AKT pathway in breast cancer cells. However, these studies investigated B7-H1 levels in total cellular proteins and not the nuclear fraction [29,30]. It is important to mention that LY294002 can also inhibit several PI3K-like kinases including mTOR, PDK2 and CK2 [45,46]. Therefore, it is possible that LY294002 is also inducing apoptosis through other pathways than PI3K/AKT and this requires further investigation.

The results of the current study demonstrate that the translocation of B7-H1 from the membrane to the nucleus could not be inhibited after PI3K/Akt pathway inhibition; rather there was a synergistic decrease in the cell surface of B7-H1 following PI3/Akt pathway inhibition and doxorubicin treatment. This suggests that the PI3K/Akt pathway is not involved in the doxorubicin-dependent downregulation of cell surface B7-H1. In conclusion, our results suggest that B7-H1 re-distribution by doxorubicin is controlled by two pathways; an AKT-dependent pathway that is dominant in the nucleus and an unknown AKT-independent pathway that is dominant in the cell surface.

The *in vivo *effect of doxorubicin on B7-H1 expression is important as cells in culture do not necessarily recapitulate the behavior of cells *in vitro*. More importantly, doxorubicin's effect on B7-H1 expression was not limited to xenotransplanted cancer cells, as similar results were observed clearly in murine cardiac tissues. These novel findings of B7-H1 downregulation in heart tissue following doxorubicin treatment may explain the cardiomyotoxicity that is reported in patients receiving this chemotherapy, beside other previously reported mechanisms [47]. Downregulation of B7-H1 in heart tissues following doxorubicin treatment may render cardiac cells a potential target for autoimmunity, which is an area for further investigation.

## Conclusions

Our findings demonstrate the presence of nuclear expression of B7-H1 in breast cancer cells and its upregulation after drug treatment and explain the previously reported immunomodulatory effect of anthracyclines on cancer cells providing a possible link between immunoresistance and chemoresistance. Finally our results suggest the use of dual combinatorial agents to inhibit B7-H1 beside chemotherapy in breast cancer patients.

## Abbreviations

APC: antigen presenting cells; CTL: cytotoxic T lymphocyte; ER: estrogen receptor; FACS: fluorescence activated cell sorting; HMGB1: High-mobility group box 1; NK: natural killer; PD-1: Programmed Death-1, PD-L1, Programmed Death Ligand-1; PR: progesterone receptor; SDA: specific doxorubicin induced apoptosis.

## Competing interests

The authors declare that they have no competing interests.

## Authors' contributions

HG designed the study, carried out the drug treatments, measurement of B7-H1 expression and annexin V, performed the immunofluorescence staining, coordinated the work and wrote the manuscript. CL separated the cell membrane proteins for Western blot. EB carried out the immunohistochemistry staining. KA carried out the immunofluorescence staining. AT (an anatomical pathologist) read and interpreted the sections. MA carried out the *in vivo *(mice) study and the siRNA cell transfection. SH carried out all the Western blot assays. PM analyzed the FACS data. AA helped in mice xenotransplanation and drug treatments. TA (a medical oncologist) participated in conceiving the study and provided the clinical data. AA conceived and supervised the study. SD (the principal investigator) wrote the proposal, conceived and supervised the study, and wrote and edited the manuscript. All authors read and approved the final manuscript.

## Supplementary Material

Additional file 1 **Supplement 1**. Inhibition of B7-H1 expression in MDA-MB-231 cells using a different specific siRNA (CD274: siRNA ID = s26548). Click here for file
