# Supplementary material for: Doxorubicin downregulates cell surface B7-H1 expression and upregulates its nuclear expression in breast cancer cells: role of B7-H1 as an anti-apoptotic molecule
Source: Breast Cancer Res. 2010 Jul 13;12(4):R48. doi: 10.1186/bcr2605 (PMC2949635; doi:10.1186/bcr2605)
Supplement: Additional file 1 — Supplement 1. Inhibition of B7-H1 expression in MDA-MB-231 cells using a different specific siRNA (CD274: siRNA ID = s26548). [file bcr2605-S1.PPT]

## Slide 1
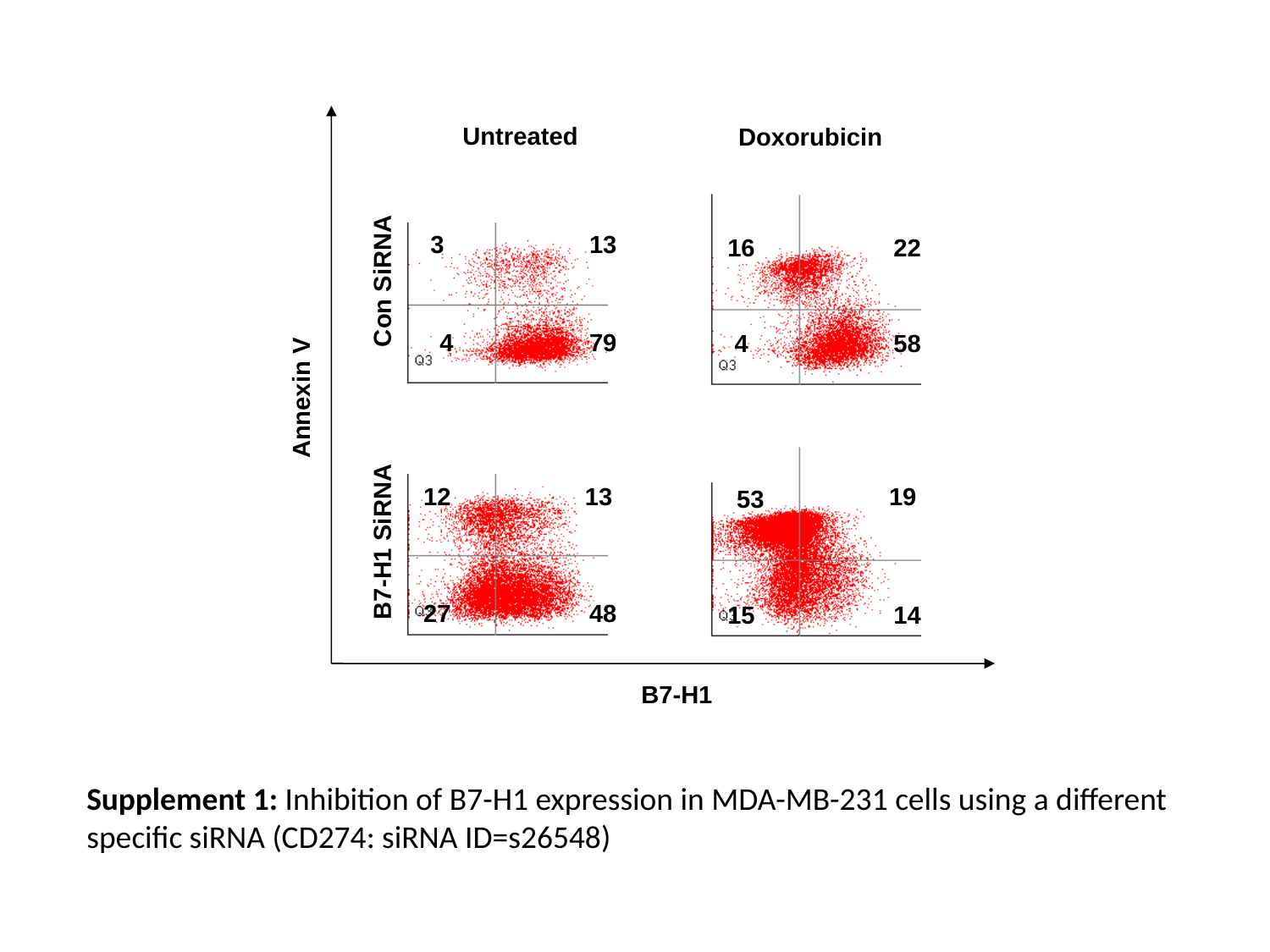

Untreated
Doxorubicin
3
13
16
22
Con SiRNA
4
79
4
58
Annexin V
12
13
19
53
B7-H1 SiRNA
27
48
15
14
B7-H1
Supplement 1: Inhibition of B7-H1 expression in MDA-MB-231 cells using a different specific siRNA (CD274: siRNA ID=s26548)
